# Supplementary material for: Impact of Serious Games on Body Composition, Physical Activity, and Dietary Change in Children and Adolescents: A Systematic Review and Meta-Analysis of Randomized Controlled Trials
Source: Nutrients. 2024 Apr 26;16(9):1290. doi: 10.3390/nu16091290 (PMC11085665; doi:10.3390/nu16091290)
Supplement: Supplementary file 1 [file nutrients-16-01290-s001.zip › Supplementary file S2.pdf]

Supplementary file S2

Table S2. Search strategies and results

(a) PubMed

Search number	Search terms	Search results
1	(Adolescent[Title/Abstract]) OR (children[Title/Abstract])	
2	(Adolescent[MeSH Terms]) OR (child[MeSH Terms])	
3	#1 OR #2	
4	(game) OR (gaming) OR (exergam*) OR (serious game) OR (video game) OR (videogame) OR (game-based) OR (gamifi*)	
5	(Video Games[MeSH Terms]) OR (Exergaming[MeSH Terms]) OR (Games, Experimental[MeSH Terms]) OR (Gamification[MeSH Terms])	
6	#4 OR #5	
7	(BMI) OR (weight) OR (physical activity) OR [64] OR (exercise) OR (eating) OR (food) OR (dietary habit*) OR (nutrition* knowledge)	
8	(Body Mass Index[MeSH Terms]) OR (Body Weight[MeSH Terms]) OR (Exercise[MeSH Terms]) OR (Food Preferences[MeSH Terms]) OR (Feeding Behavior[MeSH Terms]) OR (Diet[MeSH Terms])	
9	#7 OR #8	
10	#3 AND #6 AND #9	
11	(randomized controlled trial[Publication Type]) OR (controlled clinical trial[Publication Type]) OR (RCT) OR (randomized[Title/Abstract]) OR (trial[Title/Abstract]) OR (randomly[Title/Abstract]) OR (Clinical Trials as Topic[MeSH Terms])	
12	#3 AND #6 AND #9 AND #11	1029

(b) Web Of Science

Search number	Search terms	Search results
1	TS=(Adolescen*) OR (children)	
2	TS=(game) OR (exergame*) OR (serious game) OR (video gam*) OR (videogam*) OR (game-based) OR (gamifi*)	
3	TS=("BMI") OR ("body mass index") OR (weight) OR (physical activity) OR [64] OR (exercise) OR (eating) OR (food) OR (dietary habit*) OR (nutrition* knowledge)	
4	#1 AND #2 AND #3	
5	TS=(RCT) OR ("clinical trial") OR ("controlled trial") OR ("follow-up stud*") OR ("prospective stud*") OR ("random*") OR ("single blind*") OR ("double blind*")	
6	#1 AND #2 AND #3 AND #5	1035

(c) EMBASE

Search number	Search terms	Search results
1	Adolescent OR children	
2	'adolescent'/exp OR 'child'/exp	
3	#1 OR #2	
4	'game':ti,ab,kw OR 'exergam*':ti,ab,kw OR 'serious game':ti,ab,kw OR 'video game':ti,ab,kw OR 'videogame':ti,ab,kw OR 'game-based':ti,ab,kw OR 'gamifi*':ti,ab,kw	
5	'game'/exp OR 'video game'/exp OR 'gamification'/exp	
6	#4 OR #5	
7	'BMI' OR 'body mass index' OR 'weight' OR 'eating' OR 'food' OR 'dietary' OR 'nutrition* knowledge' OR 'sport' OR 'exercise'	
8	'body mass'/exp OR 'body weight'/exp OR 'eating habit'/exp OR 'food preference'/exp OR 'physical activity'/exp OR 'diet'/exp	

9	#7 OR #8	
10	#3 AND #6 AND #9	
11	('clinical trial'/de OR 'controlled clinical trial'/de OR 'randomized controlled trial'/de OR 'randomized controlled trial topi	
12	#3 AND #6 AND #9 AND #11	577

(d)Scopus

Search number	Search terms	Search results
1	TITLE-ABS-KEY("Adolescen*" OR "children")	
2	TITLE-ABS-KEY("game" OR "exergam*" OR "serious game" OR "video game" OR "videogame" OR game-based OR gamifi*)	
3	TITLE-ABS-KEY(BMI OR "body mass index" OR weight OR "physical activity" OR sport OR exercise OR eating OR food OR "diet" OR "food preference" OR "nutrition* knowledge")	
4	TITLE-ABS-KEY ("randomi?ed controlled trial" OR "randomi?ed clinical trial" OR "controlled clinical trial" OR randomi?ed OR rct)	
5	#1 AND #2 AND #3	
6	#1 AND #2 AND #3 AND #4	827
