# Supplementary material for: Impact of Serious Games on Body Composition, Physical Activity, and Dietary Change in Children and Adolescents: A Systematic Review and Meta-Analysis of Randomized Controlled Trials
Source: Nutrients. 2024 Apr 26;16(9):1290. doi: 10.3390/nu16091290 (PMC11085665; doi:10.3390/nu16091290)
Supplement: Supplementary file 1 [file nutrients-16-01290-s001.zip › Supplementary file S3.pdf]

Supplementary file S3: Leave-one-out sensitivity analysis

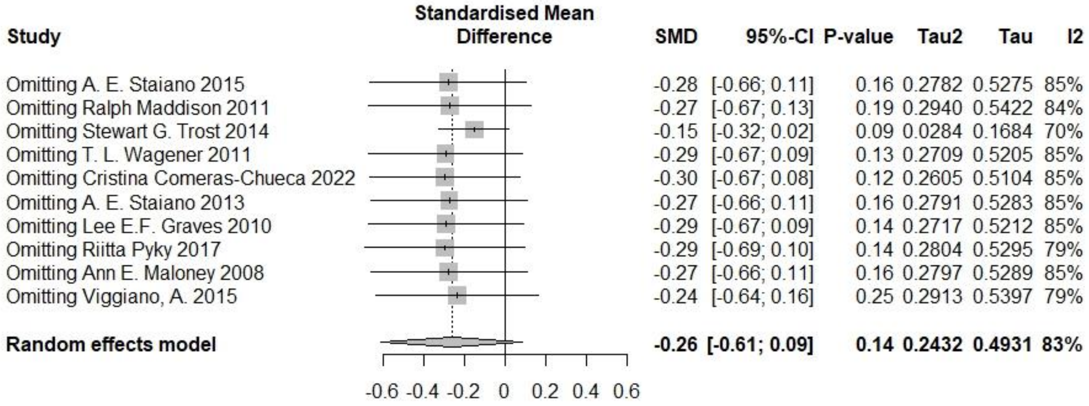

Figure S1. Sensitivity analysis for body composition

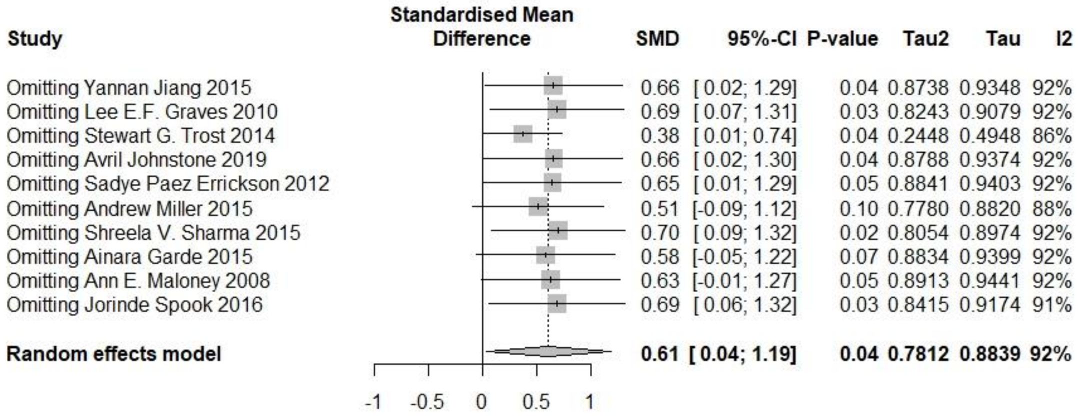

Figure S2. Sensitivity analysis for physical activity

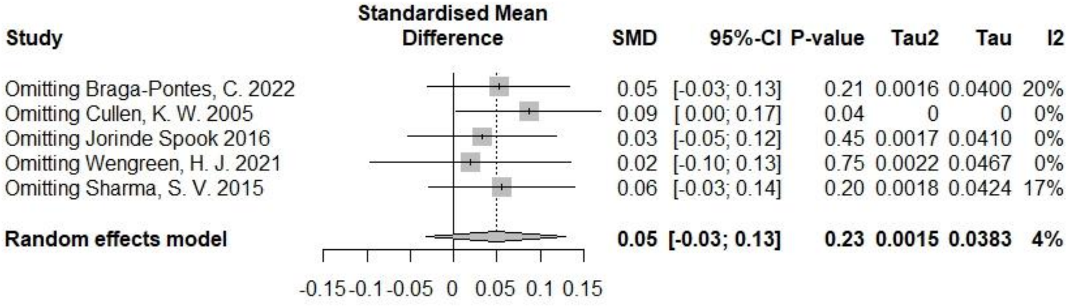

Figure S3. Sensitivity analysis for dietary change
