# Supplementary material for: Impact of Serious Games on Body Composition, Physical Activity, and Dietary Change in Children and Adolescents: A Systematic Review and Meta-Analysis of Randomized Controlled Trials
Source: Nutrients. 2024 Apr 26;16(9):1290. doi: 10.3390/nu16091290 (PMC11085665; doi:10.3390/nu16091290)
Supplement: Supplementary file 1 [file nutrients-16-01290-s001.zip › Supplementary file S4.pdf]

Supplementary file S4: Assessment of publication bias by funnel plot

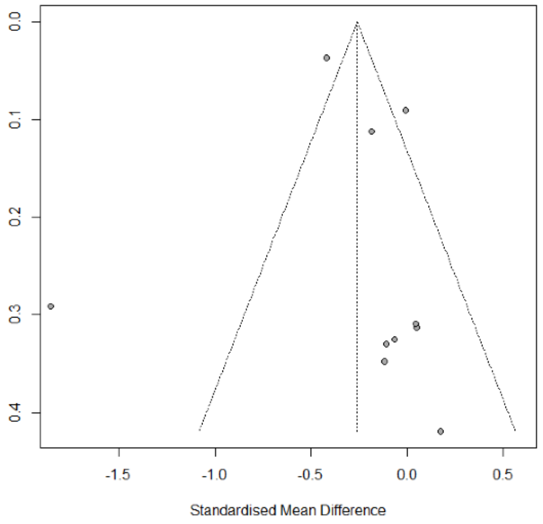

Figure S4. Publication bias for body composition

Linear regression test of funnel plot asymmetry

Test result: t = 0.70, df = 8, p-value = 0.5037

Sample estimates:

bias	se.bias	intercept	se.intercept
0.7574	1.0817	-0.3980	0.1068

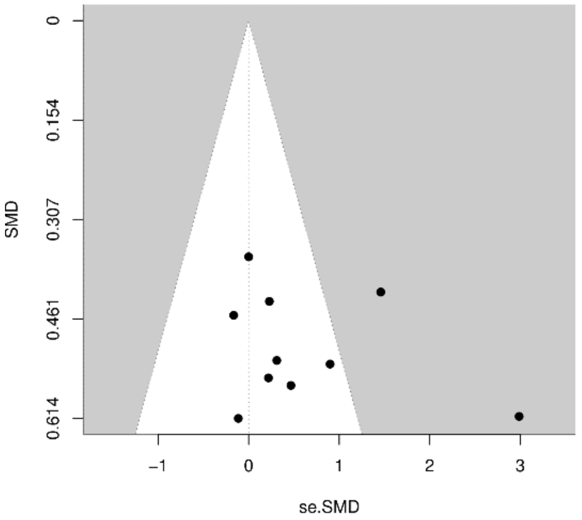

Figure S5. Publication bias for physical activity

Linear regression test of funnel plot asymmetry

Test result: t = 0.93, df = 8, p-value = 0.3791

Sample estimates:

bias	se.bias	intercept	se.intercept
3.0655	3.2930	-0.1827	0.7342
